# Chemsex drugs: More than ghb, mephedrone and methamphetamine?

**DOI:** 10.1192/j.eurpsy.2021.1528

**Published:** 2021-08-13

**Authors:** J. Curto Ramos, H. Dolengevich, M.A. Morillas Romerosa, E. Mateos Pascual

**Affiliations:** 1 Mental Health Service, University Hospital La Paz, MADRID, Spain; 2 Hospital Del Henares, Mental Health Unit, Madrid, Spain; 3 La Paz Hospital, Mental Health Unit, Madrid, Spain; 4 Hospital Juan Ramón Jiménez, Mental Health Unit, huelva, Spain

**Keywords:** chemsex, MSM, sexuality

## Abstract

**Introduction:**

The intentional use of drugs before or during sexual intercourse (chemsex), due to its impact on mental health, is a phenomenon of high importance in men who have sex with men. The main drugs usually described in chemsex related research are methamphetamine, mephedrone and GHB/GBL.

**Objectives:**

We present a narrative review of the evidence about the mechanisms of action of different drugs used in chemsex context.

**Methods:**

Narrative Review.

**Results:**

Different drugs have been associated with chemsex use: stimulants such as cocaine, stimulants with empathogenic properties such as mephedrone, methamphetamine, MDMA; stimulants with a psychedelic effect such as 2C-B; depressants such as GHB/GBL and ethyl chloride; and dissociative hallucinogens such as ketamine.

**Conclusions:**

Classical chemsex research includes only mephedrone, metanphetamine and GHB as “chemsex drugs”. Recently, different drugs have been described associated with chemsex practice. Clinicians may encounter polydrug chemsex users and the different mecanisms of action, mental health problems related to every drug and polydrug use must be takek into account.

